# Intracranial Blood Flow Velocity in Patients with **β**-Thalassemia Intermedia Using Transcranial Doppler Sonography: A Case-Control Study

**DOI:** 10.1155/2012/798296

**Published:** 2011-12-29

**Authors:** Nahid Ashjazadeh, Sajad Emami, Peyman Petramfar, Ehsan Yaghoubi, Mehran Karimi

**Affiliations:** ^1^Shiraz Neuroscience Research Center, Department of Neurology, Shiraz University of Medical Sciences, Shiraz, Iran; ^2^Hematology Research Center, Shiraz University of Medical Sciences, Shiraz, Iran

## Abstract

*Introduction*. Patients with **β**-thalassemia intermedia have a higher incidence of thromboembolic events compared to the general population. Previous studies have shown that patients with sickle cell disease, who are also prone to ischemic events, have higher intracranial arterial blood flow velocities measured by transcranial Doppler sonography (TCD). The aim of this study is to evaluate intracranial arterial flow velocities in patients with **β**-thalassemia intermedia and compare the results with those found in healthy subjects. *Methods*. Sixty-four patients with **β**-thalassemia intermedia and 30 healthy subjects underwent transcranial Doppler sonography. *Results*. Significantly higher flow velocities were found in intracranial arteries of patients compared to controls (*P* = 0.001). Previously splenectomized patients with thrombocytosis showed higher flow velocities than nonsplenectomized patients without thrombosis. *Conclusion*. The increased flow velocities in patients with **β**-thalassemia intermedia may point to a higher risk of ischemic events. Preventive measures such as blood transfusion or antiplatelet treatment may be beneficial in these patients.

## 1. Introduction

Patients with *β*-thalassemia intermedia (B-TI) seem to show higher rates of thromboembolic events than individuals without thalassemia or patients with *β*-thalassemia major,in particular if they have been splenectomized [1]. It is estimated that 4% of patients with *β*-thalassemia intermedia will experience a thromboembolic event [2]. Previous splenectomy and thrombocytosis and/or platelet abnormalities are major factors associated with thromboembolic events in patients with *β*-thalassemia intermedia [1, 3, 4], and ischemic stroke is increasingly recognized as one of the most devastating complications of this disease [5].

Ischemic stroke is also a known complication of sickle cell disease [6]. In a prospective study in patients with this condition, higher blood flow velocity in the intracranial arteries was associated with a higher risk of ischemic stroke [7]. The stroke prevention trial in sickle cell anemia (STOP) has indicated a role for transcranial Doppler sonography (TCD) in measuring intracranial arterial flow velocities to identify sickle cell patients at a high risk of ischemic stroke [8]. TCD measurement of intracranial flow velocities is of aid in deciding when to start blood transfusion in these patients to reduce the risk of ischemic stroke [8, 9].

An association between TCD findings and stroke risk has been confirmed in sickle cell disease; however, as far as we know, no studies have been conducted to evaluate TCD findings in patients with *β*-thalassemia intermedia, another high-risk group for ischemic stroke. In the present study, we compared the intracranial arterial flow velocities of *β*-thalassemia intermedia patients with those of healthy subjects.

## 2. Patients and Methods

This is a case-control study conducted in a tertiary outpatient clinic affiliated with Shiraz University of Medical Sciences, Southern Iran, for a period of one year during 2009. Consecutive patients older than 15 years of age with confirmed *β*-thalassemia intermedia by complete blood count and hemoglobin electrophoresis who were referred to an outpatient thalassemia clinic enrolled in the study. Diagnosis of B-TI was based on complete blood count, hemoglobin electrophoresis, and initial hemoglobin (Hb) level of 7 gr/dL, and age of diagnosed anemia was after 2. All of them were transfusion independent. Patients were recruited at a routine follow-up visit with a hematologist in the clinic. Exclusion criteria were a history of diabetes mellitus, hypertension, ischemic heart disease, thrombosis, previous cerebrovascular disease, sickle cell anemia, or inadequate temporal window for TCD. The study was approved by the Ethics Committee of Shiraz University of Medical Sciences (no. 2885), and written informed consent to participate was obtained from all patients or their first-degree families. All patients were receiving folic acid (5 mg/day) and hydroxyurea (8–15 mg/kg/day).

For each patient, we completed a data collection form that included age, sex, place of residence, prior splenectomy, prior transfusion, history of thrombosis, previous stroke or transient ischemic attack, and laboratory information (complete blood cell count, ferritin, blood urea nitrogen, creatinine, alanine aminotransferase, aspartate aminotransferase, alkaline phosphatase, and albumin). Thrombocytosis was defined as a platelet count >500,000/dL.

Transcranial Doppler ultrasound was carried out in all patients. All TCD studies were performed by one investigator using a Legend TC22 transcranial Doppler ultrasound unit (Bristol, UK) with a 2-MHz transducer. The two middle cerebral arteries (MCAs), anterior cerebral arteries (ACAs), posterior cerebral arteries (PCAs), and the terminal internal carotid arteries were insonated using a temporal window approach (Eleven patients had poor temporal window who were excluded from the study). The basilar artery (BA) and vertebral arteries were examined through a suboccipital approach in sitting position. The highest mean flow velocity of each artery was recorded separately.

The control group consisted of 30 sex/age-matched subjects with no known hematologic disease. The same exclusion criteria as for the patients were used for the controls. All the control subjects underwent TCD examination with the same protocol.

The Mann-Whitney *U* test, Pearson correlation coefficient (*r*), and *t*-test were used for comparison of the variables between groups. Results are expressed as percentages and absolute frequencies, where appropriate. Descriptive results are presented as the mean ± standard deviation (SD). *P* values <0.05 were considered significant. Statistical analyses were performed in SPSS version 15.0 (SPSS, Chicago, Ill, USA).

## 3. Results

 After applying the inclusion and exclusion criteria, 64 patients with *β*-thalassemia intermedia and 30 healthy subjects were recruited. There were no significant differences between patients and controls according to sex (male: 40.6% versus 53.3%; *P* = 0.251) or age (23.6 ± 5.2 versus 25.4 ± 5.4; *P* = 0.001). Mean velocities of all mentioned vessels were measured in all patients and control subjects, and no missing vessel was detected. Among the patients, 54.7% (35/64) had undergone splenectomy, and 9.4% (6/64) had received a blood transfusion once or twice a year before the study. None of the control subjects had received transfusion. In patients, mean Hb level was 9.3 ± 1.2 g/dL (range 6.9–12.3), mean white blood cell count was 8873 ± 2004/dL (range 4900–13800), and mean platelet count was 523 × 10^3^ ± 219 × 10^3^/dL (range 188 × 10^3^–1035 × 10^3^). There were no significant differences in age, white blood cell count, or Hb level between splenectomized and nonsplenectomized patients (*P* > 0.05). Mean platelet count was significantly higher in patients who had undergone splenectomy (696 × 10^3^ ± 129 × 10^3^/dL versus 315 × 10^3^ ± 78 × 10^3^/dL; *P* = 0.001). All the splenectomized patients had thrombocytosis, and none of the nonsplenectomized patients had thrombocytosis. Platelet count correlated with blood flow velocity in the right MCA (*r* = 0.291, *P* = 0.020), left MCA (*r* = 0.366, *P* = 0.003), left ACA (*r* = 0.258, *P* = 0.040), right PCA (*r* = 0.270, *P* = 0.031), left PCA (*r* = 0.267, *P* = 0.033), and BA (*r* = 0.300, *P* = 0.016). There were no correlations between platelet count and blood flow velocities of the other arteries (*P* > 0.05). Mean intracranial arterial flow velocities in *β*-thalassemia intermedia patients with or without splenectomy are shown in Table 1. There were no significant differences in white blood cell count, Hb levels, or platelet count between patients who had undergone transfusion and those who had not (*P* > 0.05).

 Comparison of the mean intracranial artery flow velocities between patients and controls showed significantly higher velocities in all intracranial arteries of patients (*P* = 0.001). Mean flow velocities recorded in patients and controls are shown in Table 2.

## 4. Discussion

In this study, higher blood flow velocities were found in all intracranial arteries of our patients. Flow velocity was higher in most arteries of splenectomized patients compared to nonsplenectomized patients, especially in the anterior circulation. In addition, there was a correlation between platelet count and flow velocities.

Numerous studies in sickle cell anemia have evaluated the role of TCD as a screening tool to identify and followup patients at high risk of ischemic stroke [7–10]. TCD can also detect asymptomatic cerebrovascular disease in patients with sickle *β*-thalassemia [11], and the findings on TCD study show a good correlation with cerebral angiography [12]. However, no such studies have been performed in *β*-thalassemia intermedia, another hematologic disease in which there is a substantial associated risk of ischemic stroke. Screening of asymptomatic *β*-thalassemia intermedia patients by TCD has not been reported previously, and the impact of this measure on further stroke remains to be defined. TI patients are prone to thromboembolic event, especially those patients associated with splenectonomy and thrombocytosis [2, 3].

There is growing evidence that increased intracranial arterial flow velocities associate with a higher risk of ischemic stroke [7, 13]. The recommendation for chronic red blood cell transfusion in patients with high stroke risk is based on findings from the STOP trial, in which patients with high intracranial arterial flow velocities showed a 90% reduction in stroke rate following this treatment [8]. Patients with *β*-thalassemia intermedia do not regularly receive blood transfusion, and they have a higher risk of ischemic stroke than *β*-thalassemia major patients, who are often treated with transfusion [3]. In addition, a higher rate of thromboembolic events occurs in splenectomized *β*-thalassemia intermedia patients [3, 14], who usually have a higher platelet count. The presence of a high flow velocity in patients with *β*-thalassemia intermedia may herald a cerebrovascular event and indicate a need for special attention. The association of flow velocity with splenectomy and platelet count suggests that these two factors should also be taken into account when interpreting stroke risk in a patient with *β*-thalassemia intermedia.

This study has some limitations. Patients were not prospectively followedup to clarify the impact of higher blood flow velocities on the risk of ischemic stroke. The interpretation presented here is based on previous studies, mainly in sickle cell disease, which showed a higher risk of stroke in association with higher flow velocities measured by TCD. Furthermore, we did not measure flow velocities before and after blood transfusion to estimate the impact of this measure on reducing velocities.

In conclusion, in this first study evaluating TCD findings in *β*-thalassemia intermedia patients, higher intracranial arterial blood flow velocities were found in comparison to normal subjects. These findings may indicate that these patients are at a higher risk of ischemic events and that preventive measures, such as blood transfusion or antiplatelet drug administration, could be beneficial. Nonetheless, prospective randomized clinical trials would be needed to establish recommendations in this regard.

## Figures and Tables

**Table 1:** Mean intracranial arterial flow velocities in *β*-thalassemia intermedia patients with or without splenectomy.

	Without splenectomy	With splenectomy	*P* value
Left ICA	84.6 ± 24.4	94.1 ± 20.7	0.097
Right ICA	83.1 ± 25.6	86.5 ± 25.1	0.538
Left MCA	66.6 ± 26.9	87.9 ± 23.9	0.001
Right MCA	71.3 ± 28.2	85.8 ± 22.7	0.026
Left ACA	58.2 ± 19.7	73.4 ± 18.3	0.002
Right ACA	57.3 ± 19.0	69.2 ± 18.2	0.013
Left PCA	35.1 ± 11.7	42.1 ± 11.6	0.021
Right PCA	34.8 ± 10.8	41.7 ± 14.3	0.037
Left vertebral artery	52.2 ± 15.0	58.3 ± 14.5	0.102
Right vertebral artery	55.6 ± 15.3	62.4 ± 17.5	0.106
Basilar artery	66.1 ± 17.2	76.7 ± 16.6	0.016

ICA, internal carotid artery; MCA, middle cerebral artery; ACA, anterior cerebral artery; PCA, posterior cerebral artery.

**Table 2:** Mean intracranial arterial flow velocities in *β*-thalassemia intermedia patients and healthy controls.

	Patients	Healthy subjects	*P* value
Left ICA	89.8 ± 22.8	52.1 ± 8.6	0.001
Right ICA	84.9 ± 21.7	50.0 ± 8.4	0.001
Left MCA	78.3 ± 27.3	49.4 ± 9.1	0.001
Right MCA	79.3 ± 26.2	48.7 ± 7.6	0.001
Left ACA	66.5 ± 20.3	44.4 ± 9.5	0.001
Right ACA	63.8 ± 19.4	42.5 ± 7.8	0.001
Left PCA	38.9 ± 12.1	29.7 ± 6.6	0.001
Right PCA	38.6 ± 13.2	27.3 ± 6.3	0.001
Left vertebral artery	55.5 ± 14.9	29.5 ± 4.6	0.001
Right vertebral artery	59.3 ± 16.8	29.3 ± 4.6	0.001
Basilar artery	71.9 ± 17.6	47.1 ± 8.2	0.001

ICA, internal carotid artery; MCA, middle cerebral artery; ACA, anterior cerebral artery; PCA, posterior cerebral artery.
